# Editorial for the Special Issue on Fabrication, Characterization and Application of Organic/Inorganic Film Membranes and Advanced Materials (Volume II)

**DOI:** 10.3390/membranes16030105

**Published:** 2026-03-17

**Authors:** Elena Kalinina

**Affiliations:** 1 Laboratory of Complex Electrophysic Investigations, Institute of Electrophysics, Ural Branch of the Russia Academy of Sciences, 620016 Yekaterinburg, Russia; jelen456@yandex.ru; Tel.: +7-343-267-87-82; 2 Department of Physical and Inorganic Chemistry, Institute of Natural Sciences and Mathematics, Ural Federal University, 620002 Yekaterinburg, Russia

The Special Issue ‘Fabrication, Characterization and Application of Organic/Inorganic Film Membranes and Advanced Materials (Volume II)’ includes 13 articles covering various aspects of the formation and application of organic and inorganic membranes and various membrane-based devices. The publications presented in the Special Issue cover a wide range of areas, including:

- Solid-state electrolyte membranes (Contributor 1, Contributor 2, Contributor 5, Contributor 9, Contributor 11) used in the development of solid oxide fuel cells (SOFCs), oxygen pumps, and Li-ion batteries;
- Electromembrane technologies and polymer ion-exchange membranes (Contributor 3, Contributor 12);
- Ultrafiltration membranes and separation polymer membranes (Contributor 6, Contributor 7, Contributor 8, Contributor 10, Contributor 13);
- Microporous membrane gas separators (Contributor 4).

Experimental studies are published in this Special Issue, and theoretical approaches have been developed. The papers describe the application of technologies such as reactive magnetron sputtering, electrophoretic deposition (EPD), the method for reactive metal evaporation in hollow cathode arc discharge, the method for directed melt crystallization followed by mechanical separation of the ingot into individual crystalline membranes, solution polymer film technology, including polymer synthesis and the introduction of modifying nanoparticles, glass melt casting technology, ceramic technology, surface micromachining technology, and the method for carbonizing coconut fiber membrane.

In the work of Andrey Solovyev et al. (Contributor 1), the comparison of the properties of single SOFCs with different thin-film solid electrolytes was carried out with a single-layer SDC electrolyte with a thickness of 5.5 μm, as well as with the multilayer SDC/YSZ/SDC electrolyte with blocking intermediate YSZ layer thicknesses of 0.5, 1 and 1.5 μm and SDC layer thicknesses of 3 and 1 μm. To form solid electrolyte layers, the technology for reactive magnetron sputtering of solid electrolyte layers onto cermet substrates was used, followed by annealing at temperatures up to 1300 °C. The microstructural and morphological properties of the films, as well as their phase composition, were then studied. The performance of the obtained SOFCs was tested in the temperature range of 500–800 °C. For the cell with the single-layer SDC electrolyte, the specific power of 651 mW/cm^2^ was achieved at 650 °C with an open-circuit voltage (OCV) of 0.8 V. The use of the YSZ blocking interlayer led to an increase in the OCV to 1.1 V. Optimization of the thickness ratio of the electrolyte layers in the SOFC with the multilayer electrolyte made it possible to achieve a specific power value 2263 and 1132 mW/cm^2^ at 800 and 650 °C, respectively. The optimal ratio of the thicknesses of the SDC/YSZ/SDC layers was 3/1/1 μm.

The work of Efim Lyalin et al. (Contributor 2) is devoted to a promising direction—the production of thin-film lithium-ion solid electrolytes for all-solid-state batteries (ASSB), which are currently being intensively developed [1]. Li-ion solid electrolyte films of Li_7_La_3_Zr_2_O_12_ (LLZ) were obtained for the first time using the electrophoretic deposition method on Ni and Ti substrates followed by annealing in air and Ar. It was established that annealing of deposited LLZ films at 400 °C was accompanied by a phase transition from tetragonal to cubic modification of the LLZ solid electrolyte. Heat treatment of the deposited LLZ films in air at 300 °C and above caused the formation of the secondary phases of La_2_Zr_2_O_7_ and fibrous particles of Li_2_CO_3_ due to the chemical interaction of the LLZ with the components of the air environment. The formation of the secondary fibrous phase was also accompanied by an increase in film thickness from 32 to 104 μm with annealing temperature growth to 500 °C. The conductivity of LLZ films of 10^−10^…10^−7^ S/cm was achieved at 100 and 200 °C, respectively.

In the work of Aminat Uzdenova et al. (Contributor 3), theoretical approaches to the analysis of electromembrane systems applicable to solving an actual problem such as water desalination were developed [2]. The study was conducted to determine the possibilities for increasing the efficiency of electromembrane desalination by installing a rectangular flow separator, which creates conditions for reducing concentration polarization and diffusion limitations near the ion-exchange membrane by improving the mixing of the solution in the solution flow. Theoretical study of the mass transfer process in the electromembrane system was carried out using a two-dimensional mathematical model under the conditions of the development of Karman vortex streets. The authors examined the separation of vortices relative to the separator in the core of the solution flow during flow near ion-exchange membranes, which reduces the concentration polarization and accelerates the transport of ions in the salt solution. The mathematical model was developed based on the joint solution of the Nernst–Planck and Navier–Stokes equations using Comsol Multiphysics^®^ 6.0 software.

Promising materials in the form of coatings based on silicon carbonitride are of interest in the field of microporous membrane gas separators [3]. Plasma-Enhanced Chemical Vapor Deposition (PECVD) technology provides wide-ranging technological possibilities for obtaining such coatings with various physical, chemical and mechanical properties. Andrey Menshakov et al. (Contributor 4) conducted a study on the formation of nanocomposite TiSiCN coatings up to 15 µm thick using the technology of reactive evaporation of titanium in the hollow cathode discharge arc. The coating was formed as a result of the reaction of titanium with the gas reaction medium (mixture of Ar/C_2_H_2_/N_2_ gases). A liquid organosilicon precursor, hexamethyldisilazane (HMDS, [(CH_3_)_3_Si]_2_NH), was used as Si source. The possibility of regulating the chemical composition and microstructure of the coatings, as well as their microhardness, by varying the deposition rate, the composition of the vapor–gas mixture, and the discharge current was demonstrated. The conditions of TiSiCN coatings production with high microhardness (up to 42 GPa) were optimized.

The development of SOFC technology is of interest from the point of view of direct conversion of chemical energy of fuel into electrical energy with high energy efficiency [4]. High temperatures of electrochemical energy conversion offer widespread possibilities for using various fuels, including pure hydrogen and hydrocarbon fuels. Electrolyte membranes based on doped ZrO_2_ are widely used in the development of high-temperature SOFCs [5]. In the work of Dmitrii Agarkov et al. (Contributor 5), the features of the influence of high-temperature annealing in air on the phase stability of the single-crystal solid solution (ZrO_2_)_0.99−x_(Sc_2_O_3_)_x_(R_2_O_3_)_0.01_ doped with rare earth elements R = Y, Gd, Tb, and Yb at x = 0.08–0.10 was investigated. Single-crystal solid electrolyte membrane samples were obtained using directional crystallization from a melt in the cooled crucible, followed by mechanical separation of the ingot into individual crystalline membranes. The authors determined the optimal membrane composition for maximum ionic conductivity and analyzed the effect of prolonged high-temperature annealing (1000 °C, 400 h) on membrane degradation and property changes.

Haiyan Wu et al. (Contributor 6) investigated the fouling problem of CAB-GO/PES (Cocamidopropyl Betaine)—Graphene Oxide/Polyethersulfone membranes used for ultrafiltration. Concentration polarization on the membrane surface during ultrafiltration reduced its permeability, leading to decreased separation efficiency and a shortened service life [6,7]. CAB-GO nanosheets were synthesized and embedded into a Polyethersulfone polymer matrix to form the CAB-GO/PES composite membrane. The authors analyzed the composition and surface functional groups of the obtained GO and CAB-GO materials, the surface morphology and the cross-section of the PES polymer membranes. The microstructure features, hydrophilicity, mechanical properties, permeability, and separating capacity of the synthesized CAB-GO/PES composite membranes were investigated.

Galina Polotskaya et al. (Contributor 7) synthesized dense nonporous membranes Poly(2,20-biquinoline-6,60-dicarbohydrazide)-co-(bistrimelliteimide)methylene-bisan-hranylide (PHI), as well as a metal–polymer complex that included the functional groups hydrazide, amide, carboxyl, and imide. The structural, thermal and mechanical properties of the obtained membranes were investigated. Particular attention was paid to the determination of the transport properties of membranes and their selectivity using the pervaporation (PV) method of a mixture of methanol (MeOH) and dimethyl carbonate (DMC) in a wide range of concentrations, determining the relevance of the presented work. SEM studies of the film morphology were carried out, membrane deformation curves and their mechanical characteristics were determined, and thermal analyses (TGA, DTA curves) were performed.

Ultrafiltration membranes are essential in the purification and production of drinking water [8]. The membrane’s permeability, its ability to remove contaminants and its resistance to contamination are of great importance. In the work of Pfano Tshindane et al. (Contributor 8), the production of polymeric membranes by tuning of the phase separation process was demonstrated. This work is distinguished by the development of approaches to obtaining fully polymeric membranes without the use of embedded nanoparticles, which can have a toxic effect [9].

Li-ion batteries are widely used in various areas of modern technology, including portable devices and electric vehicles. Solving the safety issue of Li-ion batteries is important, especially for high-powered batteries. As a solution to this problem, the possibility of a transition from liquid electrolytes to solid electrolytes is being considered [10,11,12], especially for the solid electrolyte with NASICON structure obtained by crystallization of Li_2_O–Al_2_O_3_–GeO_2_–P_2_O_5_ glass, which exhibits a high level of Li-ion conductivity of more than 10^−4^ S/cm at room temperature. Svetlana V. Pershina et al. (Contributor 9) investigated the effect of P_2_O_5_/SiO_2_ substitution in the Li_1.5_Al_0.5_Ge_1.5_(PO_4_)_3_ solid electrolyte on the crystallization temperature, thermal stability and conductivity of the material.

The use of membrane technologies is critical in the creation of bioreactors based on micro- and ultrafiltration processes [13,14]. Jie Zhang et al. (Contributor 10) conducted a study on the efficiency of composite polymer membranes based on poly(vinylidene fluoride) (PVDF) with incorporated TiO_2_ nanoparticles. The influence of polyethylene glycol (PEG) as a dispersant and pore-forming agent on the formation of PVDF/TiO_2_ composite membranes was investigated. The functional properties of the obtained membranes were determined from the point of view of the filtration and purification processes.

Alexey Nikonov et al. (Contributor 11) investigated the possibility of forming a microtubular oxygen pump using dense composite electrode layers of La_0.8_Sr_0.2_Co_0.2_Fe_0.8_O_3−δ_ (LSCF)–Ce_0.76_Gd_0.24_O_2−δ_ (GDC) and a ceramic GDC membrane (20 μm). The use of dense electrodes significantly reduced polarization resistance by 2.5–5 times. The oxygen pump design was based on the use of GDC nanoparticles in the composite electrode. The developed system produced pure oxygen with a specific energy consumption of 2.3 kW h/m^3^ at 800 °C.

Monitoring the characteristics of proton exchange membrane cells in electrolyzers is an important task in systems for producing hydrogen from water electrolysis [15]. Chi-Yuan Lee et al. (Contributor 12) proposed the use of the integrated multichannel microsensor within a high-pressure proton-exchange membrane. The microsensor enables real-time measurements of the following parameters: current, voltage, humidity, temperature, pressure, flow and hydrogen. The proposed microsensor was implemented using surface micromachining technology.

Cheng Pan et al. (Contributor 13) examined a seawater desalination system that included solar-heated elements that evaporated and condensed water. The heater utilized a coconut fiber membrane. The authors comprehensively optimized the desalination system, including the water condensation unit, increasing its efficiency to 90%.

A distinctive feature of the publications presented in this Special Issue is the broad coverage of various technologies and methods of formation, as well as the interdisciplinary nature of the approaches used in terms of the composition, structure and functional properties of promising membrane materials.
